# The Effect of Bazedoxifene and Fulvestrant for Preventing Ovarian Hyperstimulation Syndrome: An Experimental Study

**DOI:** 10.3390/jcm14207435

**Published:** 2025-10-21

**Authors:** Fatma Ozdemir, Gokhan Acmaz, Arzu Hanim Yay, Ozge Cengiz Mat, Gozde Erturk Zararsiz, Banu Acmaz, Ipek Muderris, Sabahattin Muhtaroglu, Erol Karakas, Mevlude Inanc

**Affiliations:** 1 Department of Obstetrics and Gynecology, Faculty of Medicine, Erciyes University, 38039 Kayseri, Turkey; gokhanacmaz@gmail.com (G.A.); imuderris@gmail.com (I.M.); 2 Department of Histology and Embryology, Faculty of Medicine, Erciyes University, 38039 Kayseri, Turkey; arzuyay@erciyes.edu.tr (A.H.Y.); ozgemat@erciyes.edu.tr (O.C.M.); 3 Department of Biostatistics, Faculty of Medicine, Erciyes University, 38039 Kayseri, Turkey; gozdeerturk@erciyes.edu.tr; 4 Department of Internal Medicine, Kayseri City Hospital, 38010 Kayseri, Turkey; drbanu55@gmail.com; 5 Department of Biochemistry, Faculty of Medicine, Erciyes University, 38039 Kayseri, Turkey; muhtars@erciyes.edu.tr; 6 Private Clinic, Obstetrics and Gynecology, 38039 Kayseri, Turkey; dr.erolk@gmail.com; 7 Department of Oncology, Faculty of Medicine, Erciyes University, 38039 Kayseri, Turkey; mevludeinanc@erciyes.edu.tr

**Keywords:** fulvestrant, bazedoxifene, ovarian hyperstimulation syndrome, rat model, estrogen

## Abstract

**Background:** Ovarian hyperstimulation syndrome (OHSS) remains a major complication during controlled ovarian stimulation, particularly in women with high estradiol levels. This study aimed to investigate whether bazedoxifene or fulvestrant could be effective in preventing OHSS. **Methods:** Forty 22-day-old Wistar albino rats were randomly assigned to four groups (*n* = 10 each). Group 1 received saline (negative control). Group 2 received pregnant mare serum gonadotropin (PMSG) plus hCG (positive control). Group 3 received PMSG + hCG plus fulvestrant, and Group 4 received PMSG + hCG plus bazedoxifene. Rat weight, peritoneal fluid, follicle counts, serum estradiol and VEGF levels, and ovarian ER/VEGF immunoreactivity were evaluated. **Results:** Peritoneal fluid was absent in controls but detected in 80% of positive controls and 40% of both treatment groups. Tertiary follicles and atresia were significantly higher in OHSS rats compared to controls. Fulvestrant reduced stromal ER expression, while bazedoxifene increased it. Both drugs decreased ascites formation and weight gain. Fulvestrant treatment showed unexpectedly elevated serum estradiol levels, likely due to assay interference. **Conclusions:** Fulvestrant and bazedoxifene may reduce OHSS severity by lowering ascites formation and weight gain. These agents could be potential therapeutic candidates for OHSS with appropriate timing and dosage.

## 1. Introduction

During controlled ovarian hyperstimulation (COS), ovarian hyperstimulation syndrome (OHSS) may be detected as a complication of the stimulation protocol, and this situation may be the consequence of both in vitro fertilization (IVF) and in utero insemination (IUI) cycles [1]. OHSS can be detected in 20% of at-risk women despite various prevention and estimation approaches [2,3].

It is well-known that patients with high estradiol levels have a tendency to develop OHSS. These patients’ risk increases when human chorionic gonadotropin (HCG) becomes detectable in the serum [4]. An angiogenic molecule, vascular endothelial growth factor (VEGF), starts to increase when HCG is stimulated. It is also reported that both inflammatory mediators and VEGF play central roles in the development of OHSS [5]. Increased levels of VEGF, tumor necrosis factor-alpha (TNF-α) and malondialdehyde are detected in the tissues of patients who develop OHSS. As a result, congestion, edema and apoptosis increase significantly in the tissues of these patients [6]. In the experimental rat study conducted by Atılgan et al. in 2019, it was shown that macrolide antibiotics that suppress proinflammatory molecules such as interleukins and TNF-α may also be effective in preventing OHSS [7].

Several preventive strategies have been proposed to reduce the incidence and severity of OHSS. Current pharmacological and clinical approaches include the use of gonadotropin-releasing hormone (GnRH) antagonist protocols, dopamine agonists such as cabergoline, cycle coasting, and triggering final oocyte maturation with a GnRH agonist instead of hCG. Although these methods can significantly decrease OHSS risk, each has limitations. For example, dopamine agonists may cause gastrointestinal side effects and do not always completely prevent severe OHSS; coasting can compromise oocyte quality if extended for too long; and GnRH agonist triggering may reduce implantation rates in certain protocols. Therefore, the development of novel pharmacological agents targeting different molecular pathways remains an unmet need in preventing or treating OHSS [1,8].

As a novel selective estrogen receptor modulator (SERM), bazedoxifene is accepted as a third generation, which may show its effects like estrogen or antiestrogen depending on tissue type [9]. Bazedoxifene is different from other SERMs. The combination of bazedoxifene with estrogens does not require the addition of progesterone and provides negative feedback for follicle-stimulating hormone (FSH) secretion [10]. Bazedoxifene interacts with GP130, a part of the IL-6 receptor, and prevents IL-6 from binding to its receptor. Thus, it also shows anti-inflammatory activity [11,12].

Selective estrogen receptor modulators (SERMs) are synthetic compounds that interact with the estrogen receptor. These molecules exhibit estrogenic and antiestrogenic effects depending on tissue type. They may exhibit agonistic activity in certain tissues and estrogen antagonist activity in others [13]. Fulvestrant, unlike SERMs, exhibits antiestrogenic effects regardless of tissue type. Fulvestrant inhibits receptor dimerization, inactivates activating function 1 (AF1) and activating function 2 (AF2), decreases receptor transportation to the nuclei, and accelerates estrogen receptor degradation upon binding to estrogen receptor monomers [14].

In addition to preventing inflammation, estrogen can also mediate it. Estrogens, and particularly E2, can control the proinflammatory signals/pathways of the immune system. Excessive estrogen-producing pathologies, like OHSS, trigger the immune system by causing the overexpression of estrogen receptors (ERα and ERβ), which damages tissues and results in autoimmune disorders and malignancies. Specifically, an increased ratio of ERβ is associated with proinflammatory signatures [15]. Based on this information, it can also be said that fulvestrant exhibits anti-inflammatory activity via estrogen receptor blockade.

Considering the pathophysiological mechanisms, we aimed to investigate whether SERMs (bazedoxifene) or pure antiestrogenic drugs (fulvestrant) can be used in the treatment of OHSS.

## 2. Materials and Methods

This work was authorized by Erciyes University’s Animal Experiments Local Ethics Committee and carried out in compliance with the rules for the care and use of laboratory animals (Approval number: 18/057, Approval Date: 9 May 2018). All animal-related operations were conducted in accordance with the ARRIVE standards for in vivo research and the National Institutes of Health’s Guide for the Care and Use of Laboratory Animals. We obtained 22-day-old 40 Wistar albino rats from the Erciyes University animal center, and the research animals were treated according to guidelines developed for the use and care of animals were validated by Erciyes University. Erciyes University affirmed guideline is harmonious with the National Institutes of Health Guide for the Care and Use of Laboratory Animals, Institute of Laboratory Animal Resources, National Research Council, Washington, DC. Our laboratory uses a standard diet for feeding the rats, and animals have free access to water, and care of the rats is achieved with 12 h light and dark cycles. Care of the animals is achieved by professional laboratory technicians. Fatma Ozdemir, who was aware of every stage, took a weight reading of every rat on the 22nd day of life and right before the procedure on the 27th day. Ten rats were evaluated per group, and they were allocated randomly into four groups. During praxis, we also followed ARRIVE (Animal Research: Reporting of In Vivo Experiments) guidelines.

During the induction of the OHSS model, the selected doses and routes were based on previously validated experimental models of OHSS induction in immature rats. In these studies, daily administration of 10 IU of PMSG for four consecutive days followed by a single dose of 30 IU hCG on the fifth day successfully reproduced the characteristic ovarian changes in OHSS. The subcutaneous route was preferred to ensure gradual hormone absorption and to mimic the clinical pharmacokinetics of gonadotropins [16].

Group 1: We applied a subcutaneous (SC) 0.1 mL 0.9% saline solution at 22–26 days of life for five consecutive days. This group has not received any treatment and is called the OHSS negative group. On the 6th day of the experiment (27th day of life), rats were medicated with xylazine (10 mg/kg) and ketamine (75 mg/kg) and then operated. These doses were selected in accordance with previously established anesthetic protocols in rodents to ensure adequate anesthesia and analgesia [17].

Group 2: We used 10 rats for this group and medicated them with pregnant mare serum gonadotropin (PMSG) 10 IU SC (0.1 mL of 0.9% saline) at the 22–26 days of life for four sequential days. Then we applied an SC injection (30 IU of hCG) on the fifth day of the experiment. This group constituted the OHSS-positive group. On the 6th day of the experiment (27th day of life), rats were medicated with xylazine (10 mg/kg) and ketamine (75 mg/kg) and then operated on.

Group 3: We used 10 rats for this group and medicated them with PMSG 10 IU SC (0.1 mL of 0.9% saline) at 22–26 days of life for four sequential days. Then we applied an SC injection (30 IU of hCG) on the fifth day of the experiment. Fulvestrant (FASLODEX 250 mg/5 mL AstraZeneca Vetter Pharma-Fertigung GmbH & Co. KG, Ravensburg, Germany) was administered on the 26th and 27th days consecutively to the treatment group animals at a double dose of 5 mg/kg intraperitoneally as previously described [18]. Before administration, fulvestrant was dissolved in a small amount of ethanol and subsequently diluted with sterile 0.9% saline to ensure homogenous distribution and avoid tissue irritation. This group constitutes the fulvestrant treatment group. On the 6th day of the experiment (27th day of life), rats were medicated with xylazine (10 mg/kg) and ketamine (75 mg/kg) and then operated on.

**Figure 1:**
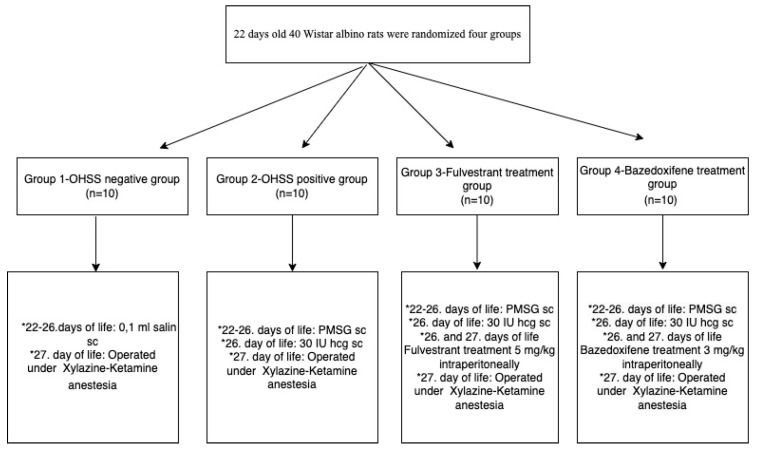
The flowchart of the study.

Group 4: We used 10 rats for this group and medicated them with PMSG 10 IU SC (0.1 mL of 0.9% saline) at 22–26 days of life for four sequential days. Then we applied an SC injection (30 IU of hCG) on the fifth day of the experiment. Bazedoxifene (Tocris 10 mg Tocris Bioscience, Bristol, BS11 9QD, UK) was administered on the 26th and 27th days consecutively to the group animals at a double dose of 3 mg/kg intraperitoneally as previously described [19]. Before administration, bazedoxifene was dissolved in dimethyl sulfoxide (DMSO) and subsequently diluted with sterile 0.9% saline to achieve complete solubilization and to minimize potential local irritation. On the 6th day of the experiment (27th day of life), rats were medicated with xylazine (10 mg/kg) and ketamine (75 mg/kg) and then operated on. The flowchart of the study is represented in Figure 1.

### 2.1. Biochemical Evaluation

Blood of rats was obtained from the tail vessel for the evaluation of serum estradiol and VEGF A. Venous blood samples taken from the tail vein were immediately centrifuged at 1200 rpm for 10 min. Then, the obtained serum sample was placed into Eppendorf tubes and stored at −80 degrees until the day of measurement. Estradiol and VEGF-A rat ELISA kits (Wuhan USCN Business Co., Ltd., Wuhan, China) were used to measure serum estradiol and VEGF-A values by thawing the serum samples after all sera were obtained. The procedures used for biochemical evaluation were performed according to previously published studies [20].

### 2.2. Peritoneal Fluid Collection

After the rats were anesthetized, peritoneal fluid was collected with a blunt injector placed in the right lower quadrant of the abdominal wall of the rat before the abdominal incision was made. Then, a midline incision was made in the lower quadrant of the abdomen, and the abdomen was entered. Peritoneal fluid detected when entering the abdomen was again collected with injectors.

### 2.3. Evaluation of Histopathology

The left and right ovaries were taken from the experimental groups to analyze histopathologic and immunohistochemical investigations. Specimens were immersed in paraffin wax after being preserved in a 10% formalin solution for 24–48 h. Each ovary was serially sectioned at 5 µm thickness [21,22,23]. Ten representative photomicrographs were taken per section at ×200 magnification. Hematoxylin–eosin (H&E) and Masson’s trichrome stain were used to stain the sections after they were cleaned in xylene and dehydrated in graded alcohol. Using a light microscope, the morphological features and differences between the groups were compared (Olympus BX51, Tokyo, Japan). Each group’s ovaries were randomly selected, and ten fields from each ovarian section were examined for ovarian follicles at various stages of development. Two observers looked over all the H&E sections.

The histopathologic and immunohistochemical evaluations were performed by two independent expert histologists blinded to the treatment groups. The final values were obtained by calculating the mean of the two observers’ results. The ovaries from each group were randomly selected prior to microscopic evaluation to minimize selection bias.

The procedures used for immunohistochemical staining, histopathological evaluation, and assessment of ovarian follicle numbers were performed according to previously published studies [21,22,23].

### 2.4. Assessment of Ovarian Follicle Numbers

The follicular types, atretic follicles, antral follicles, and corpora lutea were studied in serial sections. Follicles were classified as primary, secondary, or graafian according to the presence and size of an antrum. A pycnotic or malformed nucleus and a degraded oocyte within a degenerated follicle were observed in the atretic follicle. Every 12th section at 5 µm thickness of each ovary was analyzed to avoid double counting and counted for follicles and atretic follicles. Ten representative photomicrographs were taken per section at ×200 magnification [21]. Follicle and atretic follicle counting was performed only in follicles with a visible nucleus. The investigation employed the following previously stated diagnostic criteria [24]: The corpus luteum was made up of lutein cells, a primordial follicle was an oocyte encircled by a single layer of flattened, squamous pre-granulosa cells, a primary follicle was an oocyte encircled by a single layer of cuboidal granulosa cells, a secondary follicle had two or more layers of cuboidal granulosa cells without an antrum visible, and an antral follicle had an antral space filled with follicular fluid. The integrity of the basement membrane, cellular density, and oocyte integrity were used to assess follicular quality. Therefore, the follicles were carefully categorized into primordial, primary, preantral, antral, and atretic groups by two histologists.

### 2.5. Immunohistochemistry

The avidin-biotin-peroxidase method was used to perform immunohistochemistry on paraffin-embedded tissue specimens in accordance with the manufacturer’s instructions. Sections that were deparaffinized and 5 µm thick were rehydrated in graded alcohol and then incubated in phosphate-buffered saline (PBS) for 5 min at room temperature. To eliminate endogenous peroxidase activity, each section was treated with 3 percent hydrogen peroxide for 30 min. After that, the specimens were treated for 10 min with an Ultra V Block to prevent nonspecific staining. Next, they were incubated overnight at 4 °C with primary antibodies VEGF (ABCAM Anti-VEGF receptor 1 antibody ab2350) or ER (ER (SP1) Roche); the sections were incubated for 10 min with the biotinylated secondary antibodies. Finally, the biotinylated secondary antibodies were incubated for 10 min. The sections were treated with peroxidase-conjugated streptavidin for ten minutes following PBS washing. They were then treated for 2 min with 4′,6-diamidino-2-phenylindole (Thermo Scientific, Waltham, MA, USA), followed by Mayer’s hematoxylin counterstaining. Sections were washed with PBS and followed by peroxidase-conjugated streptavidin for 10 min. Then they were stained with 4′,6-diamidino-2-phenylindole for 2 min with subsequent counterstaining using Mayer’s hematoxylin. An Olympus DP71 digital camera coupled to an Olympus BX51 microscope was used to view immunohistochemical micrographs. Photographs were collected from ten independent microscopic fields of the tissue sections for each animal at 200 magnification to quantify the immunoreactivity intensity of the primary antibody (VEGF and ER). The intensity of immunoreactivity was evaluated using ImageJ software (ImageJ, version 1.53, Bethesda, MD, USA). We selected primordial, primary, preantral, and secondary follicles at the same magnification in each experimental group’s sections (200×). Using ImageJ software, the mean immunoreactivity intensity of VEGF and ER in an ovarian section was determined. Brown staining was recognized as VEGF or ER positive. This procedure was previously defined by Gergin et al., 2022 [25].

### 2.6. Power Analysis

The sample size was calculated by considering the hypothesis that the VEGF blood parameter differs among the experimental groups. First, a pilot study was carried out, and the effect size was estimated with samples taken from 3 mice per group. Based on this, it was calculated that at least 8 mice should be taken per group, considering the effect size (Cohen’s d) = 0.7193, alpha = 5%, and power = 90%. Considering the possible losses in the experiment, the study was carried out by taking ten mice per group and a total of 40 mice. Power analyses were applied in the statistical software PASS 11.0 (Power Analysis Statistical System, NCSS Inc., Kaysville, UT, USA).

### 2.7. Statistical Analysis

The biostatistician of the study (G. Z.) examined histograms and q-q plots. For detecting the data normality, the Shapiro–Wilk test was applied. For detecting the variance homogeneity, the Levene test was applied. Mauchly’s Test was used to evaluate the assumption of sphericity. For continuous variables (G. Z.), one-way analysis of variance (ANOVA), Welch ANOVA, or Kruskal–Wallis H tests were used to compare data among groups 1, 2, 3, and 4. The two-way repeated measures (ANOVA) test was used to evaluate the interaction between group and time. In the presence of independent categorical variables, G. Z. used the independence Pearson chi-square test. In cases where the variances were homogeneous, the Tukey test was used as a multiple comparison test. If the variances were not homogeneous, the Tamhane T2 test statistic was preferred. Bonferroni-adjusted z-tests were used in multiple comparisons of categorical variables. Moreover, a paired T-test was used to compare changes in animal weights among groups 1, 2, 3, and 4. We used our institution’s program, which is named TURCOSA (Turcosa Analytics Ltd. Co., Kayseri, Turkey, www.turcosa.com.tr, accessed on 19 October 2025). When we found a *p*-value < 0.05, it was accepted as statistically significant. All statistical analyses were performed by a biostatistician (G. Z.) who was blinded to the experimental group allocation to avoid analytical bias.

## 3. Results

Physical examinations of the rats were performed by F. O. Weights of the rats were similar for all groups before the study. After treatment, rats gained significantly more weight in the OHSS and treatment groups than in the control group. None of the rats had peritoneal fluid in the control group, but 8 of the positive control group (OHSS group) and 4 of the fulvestrant and bazedoxifene treatment group had peritoneal fluid. Comparisons of physical OHSS variables among the experimental groups are illustrated in Table 1.

H&E staining was used to assess the overall morphology of the ovarian tissues from the control and experimental groups. The ovarian cortical medulla was apparent in the control group. It was found that the follicles at all levels were active, arranged regularly, and developed normally. The corpus luteum was well-developed, and there were abundant luteal cells. Vacuolization, hemorrhage, ovarian atrophy, and disrupted cortical and medullary structures were all seen in the OHSS group. These degenerations were less observed in treatment groups than in the OHSS group. In general, many intact ovarian follicles were observed within each developmental stage.

In addition to light microscopic examination, different stages of folliculogenesis are illustrated in Table 2. The number of tertiary and atretic follicles was higher in the positive control group than in the control group. Bazedoxifene and fulvestrant treatment caused a slight decrease in the number of tertiary follicles, but the number of tertiary follicles was not significantly different in the treatment group than in the OHSS group.

**Figure 2:**
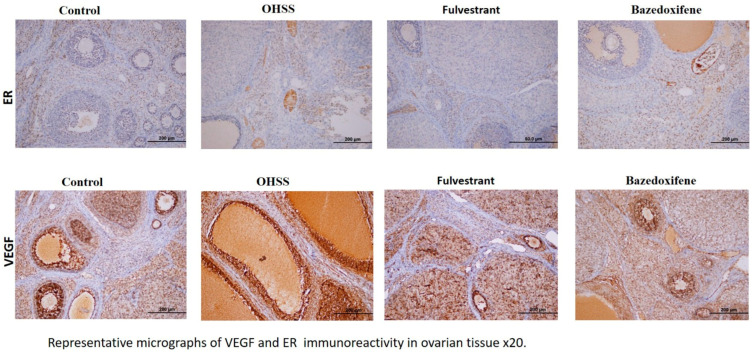
Representative micrographs of VEGF and ER immunoreactivity in ovarian tissue ×20 (*n* = 10 per group). VEGF, Vascular endothelial growth factor; ER, Estrogen receptor.

Compared with the control group, stromal ER expression was significantly upregulated in the positive control (OHSS group) and bazedoxifene treatment group. The fulvestrant treatment group showed a remarkable decrease in ER expression (Figure 2).

Blood levels of VEGF and estradiol were found to be high in the positive control (OHSS group) and treatment groups. In the fulvestrant group, stromal ER levels were found to be significantly lower than in the positive control group and the bazedoxifene group (Table 3).

**Figure 3:**
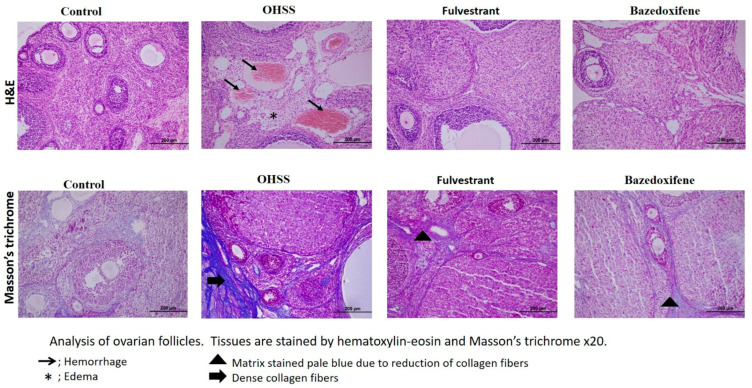
Masson’s Trichrome and Hematoxylin–eosin staining of the follicles. (*n* = 10 per group).

Investigation of the fibrotic area can be detected by distinguishing newly formed collagen fibers in ovarian tissue. To achieve this goal we used Masson’s trichrome stain. Among experimental groups, the OHSS group showed significantly increased new collagen fibers, but this increase was significantly reduced in the treatment groups. This situation is illustrated in Figure 3.

## 4. Discussion

In IVF cycles, serum estradiol levels exceeding 3500 pg/mL are considered a high-risk factor for the development of OHSS. This situation requires the use of prevention strategies to avoid OHSS development [26]. Twenty percent of at-risk women can develop OHSS, despite clinicians’ efforts to prevent OHSS through various strategies [2,3]. According to this knowledge, it is inevitable that estradiol levels become too high during COS. Therefore, we aimed to explore the therapeutic potential of a partial estrogen antagonist (bazedoxifene) and a full antagonist (fulvestrant); moreover, we investigated the effects of these drugs on serum VEGF, estradiol, rat weight, ovarian follicles, and ascites formation. Because corpus lutea can affect subsequent cycles in rats, we prefer immature rats to create an OHSS model in the rats as previously reported by Ishikawa K et al., [16].

Fulvestrant and bazedoxifene exhibit distinct but complementary pharmacological profiles that may explain their potential efficacy in reducing OHSS severity. Fulvestrant, a pure estrogen receptor antagonist, shows a long elimination half-life (~40 days) after intramuscular administration due to slow absorption and extensive distribution. It binds to ERα with high affinity, inhibits dimerisation and nuclear translocation, and promotes proteasome-mediated ER degradation, thereby reducing ER signaling and downstream VEGF expression [27].

Bazedoxifene, on the other hand, is rapidly absorbed after oral dosing, reaching Tmax within 2–3.5 h and steady state in approximately seven days, with an average elimination half-life of 25–30 h. It undergoes extensive glucuronidation and is mainly excreted unchanged in the feces (~85%), with <1% eliminated in urine. Pharmacodynamically, it acts as a tissue-selective estrogen receptor modulator—antagonistic in breast and endometrial tissues and agonistic in bone—and has also been reported to inhibit IL-6/GP130/STAT3 signaling, conferring anti-inflammatory and anti-angiogenic properties [28].

Considering these pharmacokinetic and pharmacodynamic profiles, fulvestrant’s pure ER blockade and bazedoxifene’s combined ER modulation and anti-inflammatory activity may act synergistically to attenuate VEGF-driven vascular permeability and inflammatory cascades during OHSS.

From a mechanistic perspective, fulvestrant and bazedoxifene may act on several molecular pathways involved in OHSS pathogenesis. Fulvestrant, as a pure ER antagonist, downregulates estrogen receptor expression and inhibits ER-mediated transcriptional activation. By reducing ERα signaling, it can indirectly suppress the expression of vascular endothelial growth factor (VEGF) and hypoxia-inducible factor 1-alpha (HIF-1α), thereby decreasing vascular permeability and angiogenesis [29].

In contrast, bazedoxifene acts as a selective estrogen receptor modulator and has been shown to inhibit the IL-6/GP130/STAT3 signaling cascade, which is a key proinflammatory and pro-angiogenic pathway contributing to OHSS. Inhibition of this cascade may downregulate the production of VEGF, TNF-α, and other cytokines, leading to reduced inflammatory response and endothelial leakage. Both drugs may also influence the PI3K/AKT and MAPK/ERK pathways, further contributing to decreased angiogenesis and inflammation. Together, these mechanisms may explain the observed reduction in ascites formation and VEGF levels in our treatment groups [30].

The role of VEGF and estradiol in the development of OHSS has been illustrated in the literature. The major pathophysiological mechanism underlying OHSS is acute vascular permeability (VP), which causes fluid shift from the intravascular space to the third space compartments due to exposure to exogenous and/or endogenous hCG. The hCG-induced increase in VEGF, a key regulator of VP, is the cornerstone of major pathophysiological processes in OHSS [31]. VEGF is currently recognized as the principal vasoactive and angiogenic mediator involved in OHSS. Many experimental studies for the prevention of OHSS concluded that OHSS could be prevented by suppressing VEGF and thus vascular permeability. In the study performed by Zhai et al. in 2017, it was shown that the activation of the Kisspeptin-10 pathway suppresses VEGF secretion, and this provides a significant reduction in the clinical findings of OHSS [32]. Exogenous estradiol has been shown to activate chloride channels via upregulation of cyclic adenosine monophosphate (cAMP) by using cystic fibrosis transmembrane conductance regulator in the development of OHSS. Moreover, coasting, which decreases serum estradiol levels, is used to prevent patients from OHSS [33]. Therefore, we detected the highest weight gain in the positive control group (OHSS group), and 8 of the 10 rats had ascites during physical examination. On the other hand, fulvestrant and bazedoxifene treatment significantly decreased weight gain, and 4 of the 10 rats had ascites in both the fulvestrant and bazedoxifene treatment groups during physical examination.

In the present study, the numbers of primordial, primary, and secondary follicles were similar among all experimental groups. We detected higher numbers of tertiary follicles in the positive control (OHSS group) and treatment groups. Primordial, primary, and secondary follicle developmental stages primarily depend on oocyte-derived local factors such as GDF 9 and BMP 15 and are unrelated to gonadotropin support. By contrast, the tertiary follicle stage is dependent on gonadotropic stimulation [34], and the number of tertiary follicles was found to be high in the positive control (OHSS group) and treatment groups. We detected a slight decrease in the number of tertiary follicles in treatment groups; however, there was no significant difference between the positive control (OHSS group) and treatment groups. This may be particularly important because, during IVF cycles, clinicians accept the risk of OHSS and supra-physiological hormone levels to obtain more oocytes. In the study, fulvestrant and bazedoxifene treatment decreased rat weight and ascites formation without a significant reduction in the number of tertiary follicles.

The positive control group (OHSS group) and the fulvestrant and bazedoxifene treatment groups showed significantly elevated serum VEGF levels in the study. Additionally, fulvestrant and bazedoxifene treatment decreased serum VEGF levels. Surprisingly, markedly elevated estradiol levels were observed in the fulvestrant-treated group. This unexpected result may be due to the measurement method. As is known, fulvestrant binds with a high affinity to ER and competes with estradiol. As fulvestrant and estradiol share structural similarities, cross-reactivity in immunoassays is plausible. An automated estrogen measurement assay contains only one anti-estradiol antibody binding to a single epitope on the estradiol molecule [35]. Therefore, a high estradiol level is related to fulvestrant administration, and also, we believe that fulvestrant binds to a single epitope. Thus, we found high estradiol levels in rats with fulvestrant treatment.

In the present study, we observed a significant increase in ER staining in the bazedoxifene treatment group. Contrary to this finding, the fulvestrant treatment group had significantly decreased ER staining. Moreover, we investigated ER staining on the corpus luteal site and found that the fulvestrant treatment group had significantly less ER staining than the bazedoxifene treatment group.

Oliveira CA et al. investigated the effect of fulvestrant treatment on ER. They treated rats once per week with Faslodex (10 mg/kg) and investigated ER staining. They concluded that Faslodex treatment resulted in significantly decreased ER alpha staining, but there was no effect on ER beta and androgen receptors [36]. Another mechanism of fulvestrant treatment on ER was expressed by Guan J et al. They pointed out that fulvestrant treatment slowed ER mobility to the nucleus; thus, ER turnover increased, and estrogenic activity decreased [37].

Fulvestrant is an estrogen receptor downregulator approved for the treatment of ER-positive advanced breast cancer. Large phase III clinical trials have demonstrated its efficacy and tolerability in postmenopausal women. In the FALCON study, fulvestrant 500 mg provided significantly longer progression-free survival than anastrozole [38], and the CONFIRM trial confirmed improved outcomes with the 500 mg versus 250 mg dose [39]. Its safety profile is well established; the most frequent adverse events include injection-site pain, hot flushes, nausea, headache and fatigue, with occasional asymptomatic increases in liver transaminases [40].

Bazedoxifene, a third-generation selective estrogen receptor modulator, is clinically approved for the prevention and treatment of postmenopausal osteoporosis. In a three-year randomized controlled trial, bazedoxifene significantly reduced new vertebral fracture risk compared with placebo, with a safety profile consistent with other SERMs [41]. Subsequent analyses confirmed endometrial and breast tissue neutrality and an overall favorable benefit–risk ratio. Common adverse effects include hot flushes, muscle cramps and dyspepsia, whereas an increased risk of venous thromboembolism has been reported, similar to other agents in this class [42].

Collectively, these human data indicate that fulvestrant and bazedoxifene have well-defined and generally acceptable safety profiles when used within their approved indications.

However, considering their antiestrogenic actions, any future application in assisted-reproduction settings should carefully evaluate timing and dosing to avoid potential interference with implantation or early embryonic development. Both fulvestrant and bazedoxifene are not to be used in pregnancy. In our experimental design, dosing was confined to the COS phase in immature rats, before any mating or embryo formation, precluding in utero exposure. For potential clinical translation, any application around oocyte stimulation should employ a freeze-all strategy with deferred embryo transfer until complete pharmacokinetic wash-out is achieved. This is particularly relevant given fulvestrant’s very long apparent terminal half-life after intramuscular depot injection (≈40–50 days), whereas bazedoxifene has a shorter half-life (~30 h) with predominantly fecal elimination after oral dosing. A cautious policy is to wait multiple half-lives prior to embryo transfer to avoid peri-implantation exposure. Such a strategy is consistent with OHSS-prevention practices in ART and mitigates any theoretical teratogenic risk. This study has some limitations. First, the rat model of OHSS may not fully replicate the complex pathophysiology observed in humans. Second, although the sample size was determined based on a pilot analysis of VEGF levels, other outcome parameters may require larger groups to ensure adequate statistical power. Third, the unexpectedly high estradiol levels detected in the fulvestrant group may be attributed to cross-reactivity in the estradiol immunoassay, given the structural similarity between fulvestrant and endogenous estrogen. This could have influenced the accuracy of hormone level interpretation.

Lastly, the present work was primarily designed as a morphological and immunohistochemical investigation focusing on histopathologic and biochemical alterations associated with OHSS. Additional molecular analyses such as PCR or Western blot could further strengthen the findings by elucidating gene and protein expression profiles related to estrogen receptor signaling and inflammatory pathways. We plan to address these aspects in future studies.

## 5. Conclusions

In conclusion, both fulvestrant and bazedoxifene appear to be promising agents for the prevention and treatment of OHSS in high-risk populations, provided that they are administered with appropriate timing and dosage. Both treatments were associated with reduced ascites formation and attenuated weight gain in the rat model. Although a slight decrease in the number of tertiary follicles was observed in the treatment groups, it was not statistically significant and may reflect the partial antiestrogenic activity of the agents rather than follicular suppression. These findings suggest a potential therapeutic role for fulvestrant and bazedoxifene in OHSS management, warranting further investigation in clinical settings.

## Figures and Tables

**Table 1:** Comparison of OHSS variables among the experimental groups.

OHSS Variables	Experimental Groups				*p*-Value
	Negative Control (*n* = 10)	Positive Control (*n* = 10)	Fulvestrant Treatment (*n* = 10)	Bazedoxifene Treatment (*n* = 10)	
**Rat Weight**					
**Before**	59.50 ± 1.65	58.20 ± 2.30	59.50 ± 2.84	58.90 ± 2.88	0.602
**After**	62.00 ± 2.40 **^a^**	75.90 ± 1.97 **^b^**	66.20 ± 4.05 **^c^**	67.10 ± 4.72 **^c^**	**<0.001**
**Difference**	2.50 ± 1.43 **^a^**	17.70 ± 1.64 **^b^**	6.70 ± 3.62 **^c^**	8.20 ± 5.31 **^c^**	**<0.001**
***p*-value**	**<0.001**	**<0.001**	**<0.001**	**<0.001**	
**Peritoneal fluid (present)**	0 (0.0) **^a^**	8 (80.0) **^b^**	4 (40.0) **^b^**	4 (40.0) **^b^**	**0.004**

Values are expressed as *n* (%), mean ± standard deviation or median (1st–3rd quartiles). All significant *p*-values are shown in bold. Different superscripts in the same row indicate a statistically significant difference between groups.

**Table 2:** Comparison of follicle count variables among the experimental groups.

Follicle Count	Experimental Groups				*p*-Value
	Negative Control (*n* = 10)	Positive Control (*n* = 10)	Fulvestrant Treatment (*n* = 10)	Bazedoxifene Treatment (*n* = 10)	
**Primordial**	29.50 ± 15.32	22.10 ± 9.78	16.50 ± 6.96	21.86 ± 10.96	0.114
**Primer**	50.00 ± 13.09	40.70 ± 14.69	43.60 ± 13.35	38.71 ± 12.65	0.292
**Seconder**	19.25 ± 8.28	19.70 ± 8.03	15.90 ± 6.81	17.14 ± 6.21	0.614
**Tertiary**	5.88 ± 3.60 **^a^**	15.30 ± 9.33 **^b^**	11.30 ± 6.29 **^ab^**	10.50 ± 3.13 **^ab^**	**0.019**
**Atresia**	15.00 ± 8.55 **^a^**	32.80 ± 13.64 **^b^**	20.70 ± 10.31 **^ab^**	24.93 ± 9.07 **^ab^**	**0.007**

Values are expressed as mean ± standard deviation. All significant *p*-values are shown in bold. Different superscripts in the same row indicate a statistically significant difference between groups.

**Table 3:** Comparison of ER and VEGF variables among the experimental groups.

	Experimental Groups				*p*-Value
	Negative Control	Positive Control	Fulvestrant Treatment	Bazedoxifene Treatment	
**ER variables (Stromal) Mean ± SD**	169.24 ± 3.38 **^a^**	174.32 ± 4.83 **^b^**	170.82 ± 5.00 **^a^**	176.02 ± 3.77 **^b^**	**<0.001**
**ER variables (Corpus Luteum) Mean ± SD**	172.36 ± 5.53 **^ab^**	172.79 ± 5.03 **^ab^**	171.35 ± 5.51 **^a^**	174.70 ± 5.48 **^b^**	**0.014**
**VEGF tissue**	164.69 ± 9.57 **^ab^**	160.94 ± 13.24 **^a^**	166.10 ± 11.80 **^b^**	166.06 ± 12.58 **^ab^**	**0.037**
**VEGF blood**	17.60 (15.86–26.69) **^a^**	60.97 (37.15–84.47) **^b^**	38.03 (29.80–49.12) **^b^**	53.87 (31.00–106.59) **^b^**	**0.008**
**E2 blood**	110.91 (81.67–131.62) **^a^**	137.00 (103.15–158.38) **^a^**	244.92 (179.11–358.77) **^b^**	107.62 (88.84–145.45) **^a^**	**<0.001**

SD: Standard deviation. All significant *p*-values are shown in bold. Different superscripts in the same row indicate a statistically significant difference between groups.

## Data Availability

The datasets supporting the findings of the current study are openly available in [https://zenodo.org/] at [http://doi.org/10.5281/zenodo.14059622].

## Abbreviations

The following abbreviations are used in this manuscript:

COS	Controlled ovarian hyperstimulation
OHSS	Ovarian hyperstimulation syndrome
IVF	In vitro fertilization
IUI	In utero insemination
HCG	Human chorionic gonadotropin
VEGF	Vascular endothelial growth factor
TNF-α	Tumor necrosis factor-alpha
SERM	Selective estrogen receptor modulators
AF-1	Activating function 1
AF-2	Activating function 2
ER	Estrogen receptor
Sc	subcutaneous
PMSG	Pregnant mare serum gonadotropin
H&E	Hematoxylin–eosin
PBS	Phosphate-buffered saline
PASS	Power Analysis Statistical System
ANOVA	Analysis of variance
VP	Vascular permeability
AMP	Adenosine monophosphate
